# Cryoablation of Small Renal Tumors in Patients with Solitary Kidneys: Initial Experience

**DOI:** 10.1155/2008/197324

**Published:** 2008-09-30

**Authors:** Ravi Munver, Grant I. S. Disick, Salvatore A. Lombardo, Vladislav G. Bargman, Ihor S. Sawczuk

**Affiliations:** ^1^Department of Urology, Hackensack University Medical Center, 360 Essex Street, Suite 403, Hackensack, NJ 07601, USA; ^2^Department of Urology, Touro University College of Medicine, 360 Essex Street, Suite 403, Hackensack, NJ 07601, USA; ^3^The John Theurer Cancer Center at Hackensack University Medical Center, 20 Prospect Avenue, Hackensack, NJ 07601, USA; ^4^Division of Urology, Department of Surgery, UMDNJ-New Jersey Medical School, 185 South Orange Avenue, Newark, NJ 07103, USA; ^5^Department of Urology, College of Physicians and Surgeons, Columbia University, 161 Fort Washington Avenue, New York, NY 10032, USA

## Abstract

*Introduction*. The purpose of this study was to evaluate the role of renal cryoablation in patients with solitary kidneys with the goals of tumor destruction and maximal renal parenchymal preservation. *Methods*. Eleven patients with single tumors were treated with cryoablation, of which 10 patients had solitary kidneys and 1 had a nonfunctioning contralateral kidney. All procedures were performed via an open extraperitoneal approach; ten tumors were treated with in-situ cryoablation and 1 tumor was treated with cryo-assisted partial nephrectomy. *Results*. Cryoablation was successfully performed without any preoperative complications. Mean patient age was 62.4 years (range 49–79), tumor location included: 6 (upper pole), 2 (mid-kidney), 3 (lower pole). The mean and median tumor size was 2.6 cm and 2.8 cm (range 1.2–4.3 cm), mean operative time 205 minutes (range 180–270 minutes), blood loss 98.5 ml (range 40–250 ml), and hospitalization 4.6 days (range 3–8 days). Creatinine values included: preoperative 1.43 mg/dL (range 1.2–1.9), postoperative 1.67 mg/dL (range 1.5–2.5), and nadir 1.57 mg/dL (range 1.3–2.1). All patients were followed postoperatively with magnetic resonance imaging for surveillance. At a median follow-up of 43 months, 9 patients had no evidence of recurrence, 1 patient has an enhancing indeterminate area, and 1 patient was lost to follow-up. *Conclusion*. Intermediate-term results suggest that renal cryoablation offers a feasible alternative for patients that require a maximal nephron-sparing effort with preservation of renal function and minimal risk of tumor recurrence.

## 1. INTRODUCTION

Nephron-sparing surgery (NSS) entails complete resection or destruction of a renal tumor while maximizing preservation of normal parenchymal tissue. Improvements in surgical techniques have gradually led to more widespread utilization of partial nephrectomy with acceptable postoperative morbidity and equivalent oncologic efficacy as compared to radical nephrectomy. Cryoablation is an alternative to partial nephrectomy for the treatment of renal tumors and it employs the concept of nephron-sparing surgery [1, 2].

The purpose of this study was to evaluate cryoablation as an NSS technique for the treatment of small renal masses in patients with solitary kidneys. We reviewed the application, effect on renal function, and intermediate outcomes of cryoablation in this subset of patients that require maximal renal parenchyma preservation while fulfilling the goal of tumor destruction.

## 2. MATERIALS AND METHODS

Between August 2000 and November 2004, 11 patients (9 male, 2 female) were treated with renal cryoablation for suspicious renal lesions. All patients had a single renal mass suspicious for malignancy based on radiologic imaging studies. Ten patients had a solitary kidney and 1 had a nonfunctioning contralateral kidney. Cryoablation was selected in order to offer patients a nephron-sparing procedure in cases that, due to tumor location, were not ammenable to partial nephrectomy. Patients did not receive preoperative biopsy of the renal mass lesion due to the associated risk of bleeding and renal injury in patients with solitary kidneys. Ten tumors were treated with insitu cryoablation and 1 tumor was treated with cryoassisted partial nephrectomy.

An extraperitoneal flank incision was made between the 10th and 11th ribs allowing exposure of the kidney and renal tumor. Intraoperative high-resolution renal ultrasonography with a 7.5 MHz transducer was used to establish and confirm tumor size, depth of invasion, and proximity to the renal hilar vessels and the collecting system. The renal hilar vessels were isolated but not occluded in all cases. Each tumor was biopsied with a 14gauge Tru-Cut needle prior to initiation of cyroablation.

**Figure 1:**
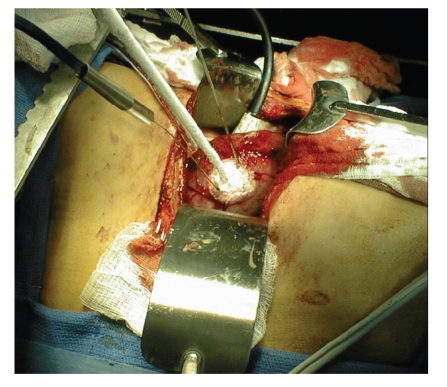
Open renal cryoablation. Cryoprobe with 2 temperature probes are seen.

Cryoablation was performed using the Cryocare surgical system (Endocare Inc., Irvine, Calif, USA). Under ultrasound guidance, 1 or 2 cryoprobes were placed directly into the identified lesion, and temperature sensor probes were placed at the periphery of each mass to provide intraoperative monitoring of adjacent renal parenchymal temperatures. In each case, the tumor margins were localized with intraoperative ultrasound, allowing for precise placement of the cryoprobes. After cryoprobe placement, the tumor was treated with 2 freeze cycles (−40C° for 10–15 minutes/cycle) (see Figure 1). Each freeze cycle was followed by an active thaw process. In the case of the partial nephrectomy, the tumor was excised with a scalpel by tracing the edge of the ice ball.

Perioperative data were evaluated including tumor size, operative time, estimated blood loss, length of hospital stay, and preoperative and postoperative creatinine.

## 3. RESULTS

Eleven patients were treated with cryoablation for 5 right renal tumors and 6 left renal tumors. The mean patient age was 62.4 years (range 49–79 years). Tumors were located in the upper pole (*n* = 6), mid-kidney (*n* = 2), and lower pole (*n* = 3) with a mean and median tumor size of 2.6 cm and 2.8 cm, respectively (range 1.2–4.3 cm). The mean operative time was 205 minutes (range 180–270 minutes), blood loss 98.5 mL (range 40–250 mL), and hospitalization 4.6 days (range 3–8 days). The procedure was successfully completed in all patients without any major intraoperative or postoperative complications.

Biopsies of the 11 lesions confirmed renal cell carcinoma (*n* = 7), oncocytoma (*n* = 2), and angiomyolipoma (*n* = 1), with one biopsy specimen that was indeterminate. The patient that underwent cryoassisted partial nephrectomy had negative margins.

The mean preoperative creatinine was 1.43 mg/dL (range 1.2–1.9) and postoperative creatinine was 1.67 mg/dL (range 1.5–2.5). The nadir creatinine was 1.57 mg/dL (range 1.3–2.1). All patients were followed postoperatively with magnetic resonance imaging (MRI) at 3–6 month intervals. Imaging these patients within the first 3 months was not performed due to our prior experience with inflammatory responses in the treated area that can lead to misinterpretation. At a median follow-up of 43 months (4–59 months), 9 patients had no evidence of recurrence, 1 patient had an indeterminate area, and 1 patient was lost to follow-up after 4 months.

## 4. DISCUSSION

Cryoablation is a minimally invasive technique that has emerged as an option for small renal masses with reduced morbidity compared to partial nephrectomy. This technology provides a nephron-sparing alternative that is curative by destruction rather than excision of the renal mass [3].

Tissue destruction from cryoablative therapy occurs from sequential freezing and thawing of tissues. Cellular destruction from the freeze process results from complex direct and indirect physiologic mechanisms, including direct physical disruption of the cellular membranes, proteins, and intracellular organelles from ice crystals. In addition, there are indirect effects such as microvascular thrombosis, osmotic dehydration, and cellular anoxia during the freeze process, which may also extend beyond the physical ice ball. The initial histologic change noted after a cryoablative procedure is coagulative necrosis. Subsequently, chronic fibrosis with collagen deposition results [2].

Early animal studies by Nakada et al. utilizing and in vivo rabbit renal cell cancer tumor model demonstrated that thermosensor-monitored renal cryosurgery produces similar outcomes to nephrectomy in terms of preventing metastatic disease [4]. Rodriguez et al. published preliminary results of series of seven patients undergoing renal cryoablation [5]. The estimated blood loss averaged 111 mL and there were no perioperative complications. Six of the 7 patients had a minimum of one follow-up computed tomography scan (mean follow-up of 14.2 months) and each of these studies demonstrated partial resolution of the lesion.

Rukstalis et al. reviewed a cohort of 29 patients that were treated with open renal cryoablation since 1996 [6]. The median preoperative renal mass size was 2.2 cm, of which 22 were solid renal masses and 7 were complex renal lesions. Five major adverse events occurred of which only one event was directly related to the procedure. At median follow-up of 16 months, 1 patient experienced a biopsy-proven local recurrence, and 91.3% of patients had a complete radiographic response with only a residual scar or small nonenhancing cyst. The authors concluded that open renal cryoablation appeared safe for the destruction of solid or complex renal masses, although rigorous radiographic, and clinical follow-up was required.

Chen et al. reported their experience with laparoscopic cryoablation of renal masses in 35 patients that underwent successful therapy with minimal postoperative complications [7]. In their series, the mean operative time was 3 hours and mean estimated blood loss was 85 mL. At 11 months of follow-up, there were no local or port site tumor recurrences. In a similar study by Gill et al., 32 patients underwent laparoscopic renal cryoablation. In this study, there were no local tumor recurrences in this group of patients [8].

Cryoablation is becoming an increasingly popular minimally invasive technique for treating renal cell carcinoma and has been shown to effectively treat renal and adrenal masses [8, 9]. This technique appears to be safe and efficacious, with recurrence rate reported as low as 6.7%, and a 5year cancer-specific survival rate of 100% for RCC [10–12].

Patients with solitary kidneys or impaired renal function may benefit from renal cryoablation as compared to partial nephrectomy for several reasons. Cryoablation may be associated with a lower risk for bleeding and may obviate the need for hilar occlusion, thus preventing the detrimental effects related to controlled ischemia. Our patients that were considered to be appropriate candidates for cryoablation did not require hilar occlusion of their solitary renal unit. While partial nephrectomy can be selectively performed without warm ischemia, the patients in our series had tumor characteristics that were not optimal for this procedure without hilar occlusion.

In our early experience with cryoablation for patients with solitary kidneys, each patient underwent an open procedure in order to minimize inadvertent injury and confirm adequate placement of the cryoprobes. Since our initial experience, we have continued our efforts to further decrease morbidity by transitioning to a laparoscopic approach for suitable tumors in this cohort of patients.

Intraoperative ultrasound was essential in delineating the intrarenal anatomy and the dimensions of the tumor [13]. Moreover, ultrasonography allows visualization of the ice ball to confirm adequate treatment of the entire lesion. In the patient that underwent cryoassisted partial nephrectomy, the tumor was excised following the freeze cycle with the intention of minimizing blood loss, as hilar occlusion was not employed. Utilization of the edge of the ice ball served as a guide for resection and facilitated complete excision of the mass with minimal blood loss.

Renal cryoablation has been reported to target kidney tumors in a precise, safe, and reproducible manner. This technology offers the ability to treat renal tumors in patients that require a maximal parenchymal sparing procedure, such as patients with solitary kidneys. Renal cryoablation allows the accurate and safe application of this surgical modality for the treatment of renal tumors with emphasis on parenchymal sparing. Additionally, the need for hilar occlusion of a solitary renal unit is completely obviated. Our intermediate follow-up data is promising in terms of both cancer control and preservation of renal function. We do not routinely perform renal biopsies following cryoablation due to the associated risks of bleeding and renal injury in patients with solitary kidneys. Instead, this practice can be reserved for patients with a suspicious enhancing area on follow-up surveillance imaging studies. If a positive biopsy is obtained in this instance, the options for surveillance, repeat cryoablation, or nephrectomy may be considered. Our results are consistent with other reports in the literature and demonstrate that renal cryoablation is a feasible technique for the management of small renal masses in patients with solitary kidneys. Additionally, combined with a laparoscopic approach, cryoablation is an attractive minimally invasive treatment option in such patients.
